# Understanding Time Series Patterns of Weight and Meal History Reports in Mobile Weight Loss Intervention Programs: Data-Driven Analysis

**DOI:** 10.2196/17521

**Published:** 2020-08-11

**Authors:** Junetae Kim, Hye Jin Kam, Youngin Kim, Yura Lee, Jae-Ho Lee

**Affiliations:** 1 Graduate School of Cancer Science and Policy National Cancer Center Goyang-si Republic of Korea; 2 Cancer Data Center National Cancer Control Institute National Cancer Center Goyang-si Republic of Korea; 3 Healthcare AI Team Healthcare Platform Center National Cancer Center Goyang-si Republic of Korea; 4 AIMMED Co, Ltd Seoul Republic of Korea; 5 Noom Inc. ‎New York, NY United States; 6 Department of Biomedical Systems Informatics Yonsei University College of Medicine Seoul Republic of Korea; 7 Department of Information Medicine Asan Medical Center University of Ulsan College of Medicine Seoul Republic of Korea; 8 Department of Emergency Medicine Asan Medical Center University of Ulsan College of Medicine Seoul Republic of Korea

**Keywords:** weight loss, self-reporting, adherence, mobile weight loss intervention, diet

## Abstract

**Background:**

Mobile apps for weight loss provide users with convenient features for recording lifestyle and health indicators; they have been widely used for weight loss recently. Previous studies in this field generally focused on the relationship between the cumulative nature of self-reported data and the results in weight loss at the end of the diet period. Therefore, we conducted an in-depth study to explore the relationships between adherence to self-reporting and weight loss outcomes during the weight reduction process.

**Objective:**

We explored the relationship between adherence to self-reporting and weight loss outcomes during the time series weight reduction process with the following 3 research questions: “How does adherence to self-reporting of body weight and meal history change over time?”, “How do weight loss outcomes depend on weight changes over time?”, and “How does adherence to the weight loss intervention change over time by gender?”

**Methods:**

We analyzed self-reported data collected weekly for 16 weeks (January 2017 to March 2018) from 684 Korean men and women who participated in a mobile weight loss intervention program provided by a mobile diet app called Noom. Analysis of variance (ANOVA) and chi-squared tests were employed to determine whether the baseline characteristics among the groups of weight loss results were different. Based on the ANOVA results and slope analysis of the trend indicating participant behavior along the time axis, we explored the relationship between adherence to self-reporting and weight loss results.

**Results:**

Adherence to self-reporting levels decreased over time, as previous studies have found. BMI change patterns (ie, absolute BMI values and change in BMI values within a week) changed over time and were characterized in 3 time series periods. The relationships between the weight loss outcome and both meal history and self-reporting patterns were gender-dependent. There was no statistical association between adherence to self-reporting and weight loss outcomes in the male participants.

**Conclusions:**

Although mobile technology has increased the convenience of self-reporting when dieting, it should be noted that technology itself is not the essence of weight loss. The in-depth understanding of the relationship between adherence to self-reporting and weight loss outcome found in this study may contribute to the development of better weight loss interventions in mobile environments.

## Introduction

Mobile apps are widely used for tracking weight loss [1-3]. A major feature of these apps is providing users with self-reporting capabilities regarding their lifestyle and health-related measurements [1-3].

Self-reporting has been recognized as a very important behavioral treatment method for weight loss [4-9] because it provides diet participants with a continuous feedback loop that can modify behaviors to achieve goals through the process of observation, evaluation, and reinforcement [5,10]. Indeed, several empirical studies have shown a positive relationship between adherence to self-reporting and satisfactory weight loss outcomes [4,5,7]. For these reasons, considerable research has been conducted regarding adherence to self-reporting in the field of weight loss research [6,8,11]. These studies commonly suggest the expectation that the use of mobile technology will improve adherence to self-reporting [6,7,12].

Compared to traditional tools, mobile technology’s portability has made it easier and more effective to monitor and control one’s weight [4,9,13-15]. In addition, the use of mobile technology such as smartphones and wearable devices enables researchers to explore new topics by collecting data that could not previously be gathered. Specifically, a representative research question related to new data may demonstrate the relationship between characteristics of adherence to self-reporting and weight loss outcomes [4,15,16]. Another research question may explore the differences in adherence to self-reporting during the diet in terms of demographic characteristics such as gender and sociocultural aspects such as race [17-22]. These studies have found that adherence to self-reporting decreases over time [4,16,21,23,24] and is dependent on demographics [17-22].

However, an in-depth understanding is lacking regarding the relationship between weight loss outcomes and the dynamics of adherence to self-reporting according to specific groups. Attempts at an in-depth understanding of this relationship is very important for several reasons. First, given that demographic characteristics affect both adherence to self-reporting and dietary approaches [17–22], researchers may gain a fundamental understanding of how certain groups respond to weight loss interventions. Second, researchers may understand the mechanism of weight changes during the weight loss process that involves a series of weight gain, loss, and regain cycles [21-23]. Ultimately, gaining an in-depth understanding of these matters will contribute to designing better weight loss programs suitable for mobile apps.

Thus, this study aimed to analyze the relationship between weight loss outcomes and the dynamics of adherence to self-reporting. To that end, we analyzed self-reported data that were aggregated on a weekly basis for 16 weeks, from January 2017 to March 2018, from 684 Korean men and women who participated in a mobile weight loss intervention program provided by a commercial smartphone app.

The following 3 research questions were explored and answered: How does adherence to self-reporting on body weight and meal history change over time? How do weight loss outcomes depend on weight changes over time? How does adherence to the weight loss intervention change over time by gender?

## Methods

### Mobile Weight Loss Intervention

The mobile intervention program for this study was delivered through a commercially available mobile coaching program called Noom [25]. During the 16-week program, users were asked to log their meals, exercise, and weight data using this app [25]. In-app articles about healthy dietary intake and promotions to encourage physical activities were provided on a daily basis [25]. The articles were adapted by physicians, clinical dietitians, and clinical psychologists and based on studies provided by the American Diabetes Association, American Heart Association, and Centers for Disease Control and Prevention.

All coaches were registered dietitians, and they were supervised weekly by a clinical psychologist. They helped users set weekly goals and provided personalized feedback based on each user’s lifestyle. Messages were sent to the users at least twice per week.

### Measurements

#### Weight Entry Trends

The frequency of weight entries was analyzed. In most health care app research programs, user adherence to a certain intervention has often been measured by the frequency of self-reporting [4,24,26,27]. Therefore, the number of body weight entries per week served as a trustworthy variable in measuring the degree of adherence to the weight loss intervention in this research [4,24,26,27].

The study participants reported their weight through the app. After reporting, weight-related menu-usage logs were automatically stored on a server. The collected usage log data were summed at weekly intervals to generate data indicating adherence to weight-reporting activity over 16 weeks, for each subject.

#### BMI Trends

BMI is often used to determine whether a person is within a healthy weight range based on their height [4,14,28]. Since this is calculated by dividing weight by height (kg/m^2^), the BMI value represents relative weight with respect to height [29,30]. In other words, BMI values indicate whether a person is “underweight,” “healthy,” “overweight,” or “obese” [4,14,28]. Because of its ease of interpretation and satisfactory explanatory power, BMI has been used instead of basic weight measurements for many diet-related studies on web and mobile weight tracking environments [4,14,25,31-33].

Participants reported their body weight daily through Noom. The daily BMI values for each participant were calculated by dividing the daily reported weights by their initially entered height values, and the daily results were averaged on a weekly basis.

BMI delta values were used to identify weekly weight fluctuation trends [26,34,35]. For this analysis, BMI delta was defined as the difference between the largest and smallest BMI values in a given week. As such, for each participant, the average weekly BMI values representing their overall obesity level and the weekly BMI deltas indicating their degree of weight fluctuation over 16 weeks were used for the statistical analysis.

#### Meal History Entry Trends

As with weight entries, the frequency of self-reported meal history entries has frequently been used in prior studies to address the level of adherence to weight loss intervention programs [4,32]. In this study, participants entered not only their main meals but also snacks in between meals. As participants recorded food items by searching for them within Noom’s food database, their eating habits and selections could be grouped in detail by food types.

These food types were categorized as green, yellow, and red groups based on their caloric density, that is, calories per gram or milliliter [29,30,36]. In general, foods in the red group have very high caloric density and include foods high in fat and oil [29,30,36]. Foods categorized as yellow have moderate caloric density; they are less fatty and oily than the red group but still not optimal [29,30,36]. Foods in the green group have very low caloric density and are comprised mostly of natural fruits and vegetables [29,30,36]. The applied food type criteria according to caloric density are described in Multimedia Appendix 1.

Variables representing food intake by food type were generated based on the aforementioned criteria. Furthermore, food intake was reported 6 times per day (ie, breakfast, morning snacks, lunch, afternoon snacks, dinner, and evening snacks). This provided a substantial amount of data regarding the changes in food intake for the 3 food groups of each participant over 16 weeks.

### Study Sample

**Figure 1:**
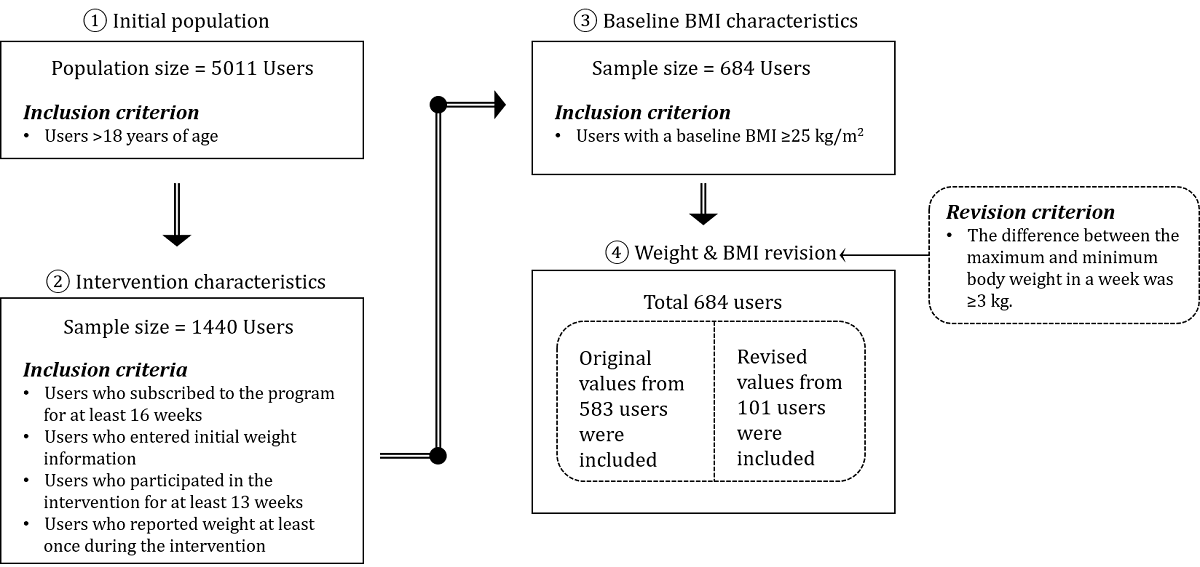
Study sample selection process.

This study was approved by the Institutional Review Board of the Asan Medical Center, Korea (No. 2017-1253). Figure 1 shows the study sample selection procedure. Initially, the study population comprised 5011 users over the age of 18 years who participated in the coaching program from January 1, 2017 to March 5, 2018 [4,37]. Of these, 1440 users were included in the study based on 4 intervention characteristics [4,9,38]. First, according to prior research, users who subscribed to the program for at least 16 weeks were included in the study [4,9,38]. Second, only users with initial weight information were included in the study, to analyze baseline weight. Third, only those who reported their weight at least once among those who participated in the intervention for at least 13 weeks were included in the study [4]. Finally, 684 overweight and obese users with BMIs >25 kg/m^2^ were selected for analysis [4,36].

Participants measured their weight on their scales and then reported the weight via the app. To prevent incorrect input that may occur when users report these weight values, certain reported values were corrected. As no previous studies provided any clear criteria for mitigating data errors, a group of three experts consisting of doctors and health care professionals set the error criteria and reviewed the data. It was assumed that there could be potential errors if the difference between the maximum weight and minimum weight per week was ≥3 kg. The daily weight and BMI values of 101 cases corresponding to this assumption were not included in the weekly averages.

### Statistical Analysis

To analyze the self-reported time-series pattern based on weight loss groups, subjects were divided into 3 groups according to recommendations from prior research [4]. The 3 groups consisted of those with weight losses <5%, of 5%-10%, and >10% [4].

Analysis of variance (ANOVA) and chi-squared tests were performed to analyze whether the baseline characteristics of the 3 groups were statistically different. Furthermore, ANOVAs along the time axis were performed to determine whether each measurement (ie, BMI, weight entry, and meal history entry trends) of the 3 groups (ie, <5%, 5%-10%, >10%) within gender were statistically different over 16 weeks. *P* values were adjusted with the false discovery rate using the Benjamini-Hochberg method, because multiple tests based on the week and groups may yield 5% false positives at the 5% significance level [39]. Finally, through slope analysis of each measurement calculated at the aggregated level, the overall time-series trends for the measurements were analyzed.

## Results

### Participant Characteristics

Table 1 shows the test results for the statistical differences in baseline characteristics between the 3 outcome groups. In the case of gender, although few men (19/218) and women (39/466) belonged to the group that lost >10% weight, there were no statistical associations among the 3 groups. For age, young participants (ie, mean 32.09 years old) seemed to lose the most weight; however, there were no statistical differences among the 3 groups. In the case of base weight status, the few obese (17/178) participants lost >10% of their weight; however, no statistical associations were found among the 3 groups.

**Table 1:** Participant characteristics for the total sample and according to outcome group.

Characteristics		Total sample	Outcome group according to level of weight loss			*P* value
			<5%	5%-10%	>10%	
**Gender, n**						
	Male	218 (31.9)^a^	126	73	19	.39^b^
	Female	466 (68.1)^a^	294	133	39	
Age (years), mean (SD)		33.31 (7.6)	33.7	32.87	32.09	.19^c^
**Baseline weight status, n**						
	Obese	178 (26)^a^	112	49	17	.62^b^
	Overweight	506 (74)^a^	308	157	41	

^a^n (%).

^b^Tested differences between the 3 groups using chi-squared tests.

^c^Tested differences between the 3 groups using analysis of variance (ANOVA).

### Pattern Analysis

#### Weight Entries

**Figure 2:**
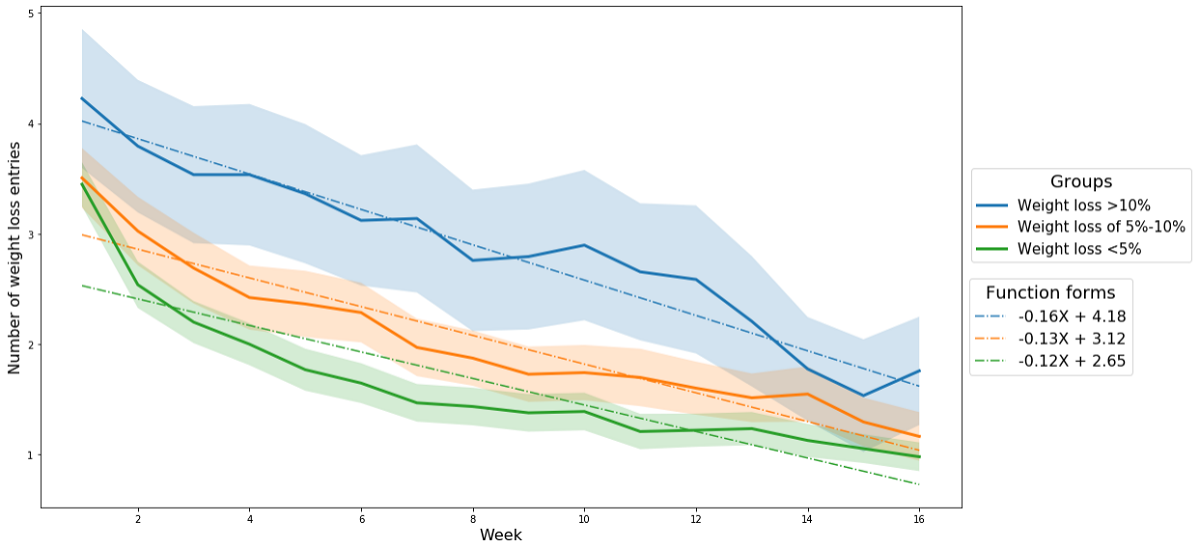
The number of weight entries on a weekly basis over 16 weeks; the colored bands show the 95% CI.

Figure 2 shows the number of weight entries reported by user each week for the 16 weeks. The number of weight entries decreased over time in all 3 groups even though the decreases in the slopes were different (ie, −0.16 for the group who lost >10% of their starting weight, −0.13 for the group who lost 5%-10%, and −0.12 for the group who lost <5%). The members of the group with >10% weight loss recorded their weight more often in Noom than the other groups (ie, the groups who lost ≤10%). This trend at the aggregated level was statistically significant throughout the entire study period (Table 2). Moreover, the statistical significance of the trend was noticeable in women (Table 2).

**Table 2:** Significance of analysis of variance (ANOVA) test results comparing the differences between groups for 16 weeks. *P* values ​​were adjusted by the false discovery rate using the Benjamini-Hochberg method, and the actual *P* values are available in Multimedia Appendix 2.

Group			Week																													
			1	2		3		4		5		6		7		8		9		10		11		12		13		14		15		16
**Weight entries**																																
	Total^a^	**^b^		***^c^	***^c^		***^c^		***^c^		***^c^		***^c^		***^c^		***^c^		***^c^		***^c^		***^c^		***^c^		***^c^		**^b^		***^c^	
	Men^a^								**^b^		**^b^								*^d^													
	Women^a^	***^c^		***^c^	***^c^		***^c^		***^c^		***^c^		***^c^		***^c^		***^c^		***^c^		***^c^		***^c^		***^c^		***^c^				***^c^	
**BMI values**																																
	Total^a^								*^d^		*^d^		**^b^		***^c^		***^c^		***^c^		***^c^		***^c^		***^c^		***^c^		***^c^		***^c^	
	Men^a^														*^d^		*^d^						**^b^		**^b^		**^b^		**^b^		*^d^	
	Women^a^								*^d^						***^c^		**^b^		**^b^		***^c^		***^c^		***^c^		***^c^		***^c^		***^c^	
**BMI delta^d^**																																
	Total^a^	***^c^		***^c^	***^c^		*^d^		***^c^		***^c^		*^d^		**^b^		*^d^				***^c^		*^d^									
	Men^a^			**^b^																												
	Women^a^	***^c^		***^c^	**^b^				***^c^		***^c^		*^d^		*^d^						***^c^		**^b^									
**Meal history entries**			** **	** **		** **		** **		** **		** **		** **		** **		** **		** **		** **		** **								
	Total^a^			*^d^	*^d^		**^b^		*^d^		***^c^		***^c^		***^c^		***^c^		***^c^		**^b^		***^c^		*^d^		**^b^		*^d^		**^b^	
	Men^a^																															
	Women^a^						*^d^		*^d^		**^b^		***^c^		**^b^		**^b^		***^c^				***^c^		*^d^		**^b^		**^b^		**^b^	

^a^When the null hypotheses were tested using ANOVA, the three groups (weight loss >10%, weight loss of 5%-10%, and weight loss <5%) had the same value.

^b^*P*<.05

^c^*P*<.01

^d^*P*<.1

^e^Defined as the difference between the maximum and minimum BMIs in a given week.

#### BMI Values

**Figure 3:**
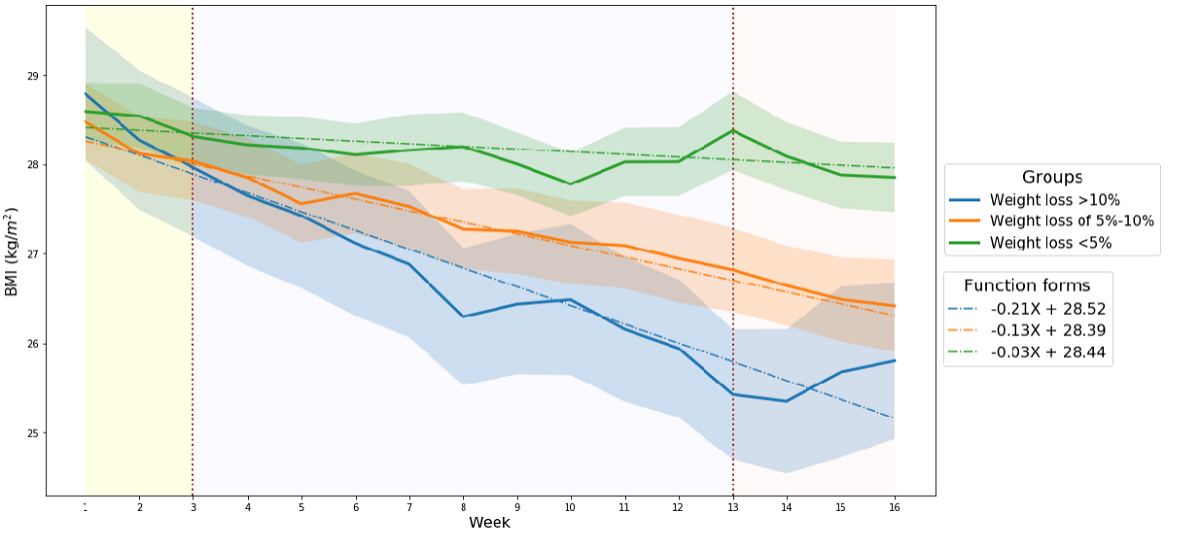
BMIs over 16 weeks according to weight loss outcome groups; the colored bands show the 95% CI.

Figure 3 shows the trend in the BMI values for the 3 groups over time at the aggregated level. The differences in the BMI values between the 3 groups was statistically significant after week 5 (Table 2). Furthermore, the statistical difference was more noticeable in women than in men (Table 2).

**Figure 4:**
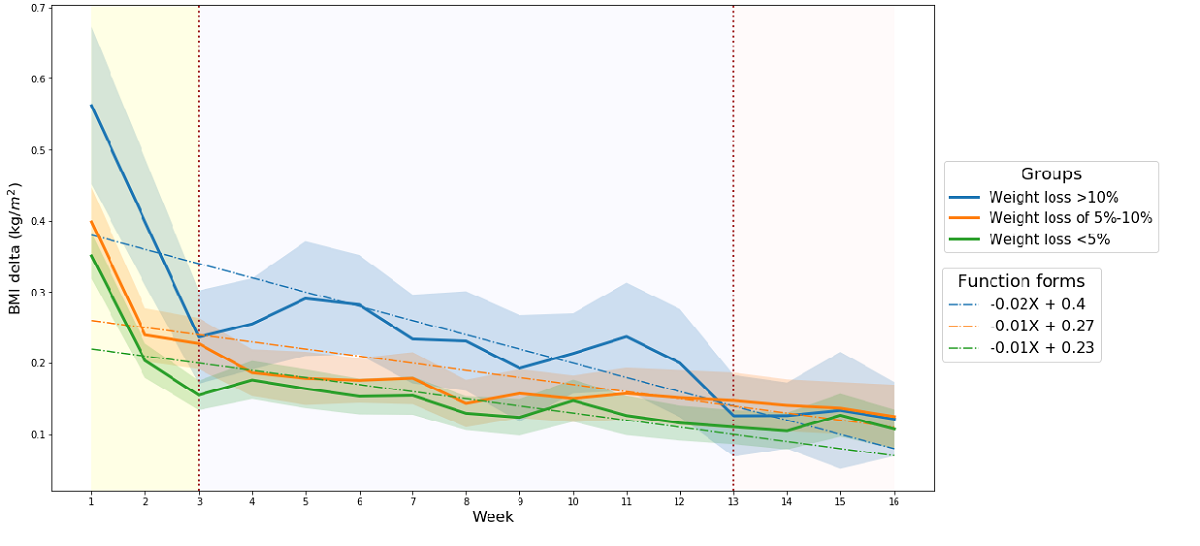
BMI delta over 16 weeks according to weight loss outcome groups; the colored bands show the 95% CI.

In all 3 groups, the BMI delta values — representing the degree of weight fluctuation — were only high during the initial 3 weeks (Figure 4). The group that lost >10% weight had larger BMI delta values during the entire study period. The difference in BMI delta values among the 3 groups was the most statistically significant during the first several weeks. However, most of the differences in values became insignificant over time (Table 2). Moreover, the BMI delta was more statistically significant in women (Table 2).

#### Meal History Entries

**Figure 5:**
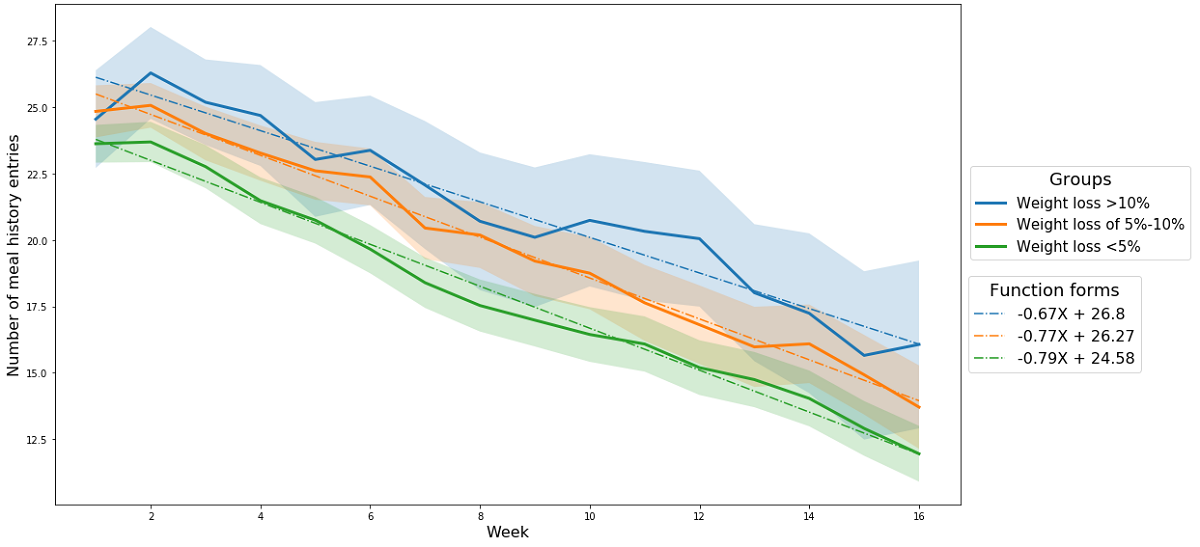
Number of meal history entries on a weekly basis over 16 weeks; the colored bands show the 95% CI.

As seen in Figure 5, the number of meal history entries decreased over time in all 3 groups (slope values of −0.67, −0.77, and −0.79 for the groups with >10% weight loss, 5%-10% weight loss, and <5% weight loss, respectively). The 2 groups that reduced their weight by ≥5% had a higher total number of meal history entries than the less successful group, and the differences in total number of meal history entries among the 3 groups were statistically significant during most of the study period (Table 2). No significant differences in the total number of meal history entries were found for men (Table 2). On the other hand, for women, significant differences were found during most of the study period (Table 2).

Table 3 shows food intakes divided into the green, yellow, and red groups for 16 weeks for women. Women had many statistical differences between the yellow and red food groups, especially at lunch and dinner. In particular, when the intake of foods in the red category was averaged over all study periods, statistical differences between the 3 groups were significant for all meal types (Table 3). However, for men, there were no statistical differences between weight loss outcome groups and food groups over the 16 weeks.

**Table 3:** Significance of analysis of variance (ANOVA) test results comparing the differences in intake amount by food group (green, yellow, and red) in women for 16 weeks. *P* values ​​were adjusted with the false discovery rate using the Benjamini-Hochberg method, and the actual *P* values are available in Multimedia Appendix 3.

Food group		Week																Avg^a^	
		1	2	3	4	5	6	7	8	9	10	11	12	13	14	15	16		
**Green**																			
	BRE^b^			**^c^															
	MOR.S^d^																		
	LUN^e^																		
	AFT.S^f^																		
	DIN^g^																		
	EVE.S^h^																		
**Yellow**																			
	BRE											**^c^							***^i^
	MOR.S																		
	LUN				**^c^					**^c^		**^c^	**^c^		**^c^				***^i^
	AFT.S																		
	DIN	**^c^			*^j^		**^c^	**^c^		*^j^		*^j^	**^c^			**^c^			***^i^
	EVE.S																		
**Red**																			
	BRE				**^c^	*^j^					*^j^	**^c^							***^i^
	MOR.S						**^c^			*^j^									**^c^
	LUN			**^c^	**^c^	*^j^	**^c^	*^j^	**^c^	*^j^	*^j^	*^j^	**^c^				**^c^		***^i^
	AFT.S		**^c^	*^j^									**^c^						***^i^
	DIN		**^c^	**^c^	**^c^	**^c^			*^j^	*^j^	**^c^	**^c^	*^j^	**^c^		**^c^			***^i^
	EVE.S	*^j^						*^j^			**^c^								***^i^

^a^Avg: grouped average of food intake over 16 weeks; the *P* value for was not adjusted by the false discovery rate using the Benjamini-Hochbert method. When the null hypotheses were tested using ANOVA, the three groups (weight loss >10%, weight loss of 5%-10%, and weight loss <5%) had the same value.

^b^BRE: breakfast.

^c^*P*<.05

^d^MOR.S: morning snack.

^e^LUN: lunch.

^f^AFT.S: afternoon snack.

^g^DIN: dinner.

^h^EVE.S: evening snack.

^i^*P*<.01

^j^*P*<.1

## Discussion

### Principal Findings

#### Decreased Level of Adherence Over Time

Previous studies showed that the degree of adherence to self-reporting in mobile weight loss intervention programs decreases over time [4,6,23]. Going one step further, this study found that adherence decreases over time regardless of weight loss outcomes. Specifically, both the total weight and meal history entries decreased gradually over 16 weeks (Figures 2 and 5). Prior studies had assumed that mobile technology utilization could overcome the low adherence to self-reporting that occurs when utilizing rudimentary paper tools during weight loss interventions [6,13]. Although mobile technology clearly makes self-reporting easier, the results of this study revealed that there is a natural tendency to lower one’s adherence to self-reporting over time regardless of weight loss outcomes.

Among the many factors that hinder long-term adherence, the most relevant to this study may be the lack of serious awareness of current health conditions [37,40,41]. In general, most people are not likely to feel any ominous threats even if they do not lose their weight right away, and they are prone to stop dieting. Therefore, to promote long-term adherence, it is necessary to provide an environment in which diet participants recognize the critical importance of weight management [42,43]. Given the lessons learned from previous diet studies that have shown that digital push notifications have no effect on adherence, reminding participants regularly about the importance of dieting may be ineffective [44,45]. Instead, there seems to be a need for further research to provide divergent strategies for people with obesity.

Conversely, it may be entirely reasonable to simply admit that humans are less committed to interventions over time, especially for slow and arduous tasks like trying to lose weight. In other words, it may be wise to avoid long-term adherence issues that are unlikely to be solved. Rather, the best practice may be to focus on understanding the characteristics of adherence with latent patterns. Recent data science algorithms may be employed to understand specific patterns of adherence. Specifically, the use of time series decomposition algorithms that eliminate the inevitable long-term declines in adherence may be used for exploring repeated patterns of adherence [46,47]. Then, demonstrating the association between the pattern and weight loss outcomes can provide new insights.

#### Characteristics of BMI Change Over Time

According to the results of this empirical analysis, BMI changes can be characterized in 3 time series periods (Figures 3 and 4). The first interval is from week 1 to week 3, where the overall BMI delta value started high and fell rapidly over time and the overall BMI value was high in the initial stages. High values for both BMI and BMI delta may indicate substantial weight fluctuations with low weight losses within a week. This phenomenon may be due to the participants' biological responses to homeostasis (ie, steady weight) [27]. It takes time for the human body to adapt to a diet (ie, limiting caloric intake and increasing energy expenditure through exercise) [27,48,49]. During this adaptation process, human weight can fluctuate based on a variety of factors, including hormones, psychology, and physiology [21-23]. Although the period of adaptation may vary depending on certain factors (eg, race, diet style), several empirical studies have found that it takes human bodies approximately 2 to 4 weeks to adapt to a diet [48,49]. Therefore, participants in this study might have experienced weight fluctuations during the dietary adaptation process period.

The second interval is between weeks 3 and 13. This interval was characterized by differences in BMI that increased over time among the outcome groups. Further, most BMI deltas were statistically different among the groups (Figures 3 and 4, Table 2). In this period, the differences in weight loss between the successful and unsuccessful diet groups were noticeable. Specifically, the successful group had high BMI deltas and low BMI values, which may indicate that the successful participants lost weight. On the other hand, the unsuccessful group during the same period had low BMI deltas and high BMI values, thereby suggesting that the unsuccessful participants rarely lost any weight at all.

The last interval is between weeks 13 and 16. This interval was characterized by no differences in BMI deltas between the groups (Figure 4). Another feature during this period was the manifestation of weight increases in the group that lost the most weight (>10%). This may indicate that the group members stopped trying to lose weight, because people do not lose weight beyond their ideal weight. From a physiological point of view, the feature may be attributed to the widely known “yo-yo” effect that commonly occurs after rapid weight loss [45-47]. The yo-yo effect is defined as a rapid weight gain after a diet. Indeed, many previous studies have shown that the majority of diet participants regain their weight over the long term [45-47]. In this study, the yo-yo effect may have occurred in participants who had dietary laxity as the mobile weight loss intervention program was nearing its end in week 16.

#### Different Patterns for Female and Male Participants

Gender effects on the differences in self-reporting adherence have often been discussed in previous studies of weight loss interventions [4,6,22]. Going one step further, this study explored the time-dependent relationships among adherence to self-reporting, gender effects, and weight loss outcomes.

Unlike female participants, male participants had little differences in the number of weight entries among the outcome groups during the 16 weeks (Table 2). Furthermore, in terms of the number of meal history entries, there were no statistical differences in the values among the outcome groups for male participants (Table 2). However, statistical differences in BMI values among the 3 groups were found around the last several weeks (Table 2).

These results suggest that there was no statistical association between adherence to self-reporting and weight loss outcomes in the male population. This contradicts some previous studies that showed positive relationships between the two [6,7,50]. Inconsistencies in the results can be addressed as follows. Considering that the tendency to self-report is influenced by various factors, such as gender and race [6,22,51], Korean adult men may not be as inclined to self-report. In other words, they may have avoided timely self-reporting regardless of the success of the diet. As self-reporting itself has no direct effect on weight loss, male participants might have been able to lose weight without consistent self-reporting if they managed their diet well and consistently exercised.

Another interesting finding in this analysis is that the male participants, unlike the female participants, demonstrated no statistical difference in the intake of food types among the 3 weight loss groups. Previous studies found that men were insensitive to food intake during their diets, while women were very sensitive [52,53]. Therefore, the results may have captured the food intake characteristics of dieting men during mobile interventions. However, it should be recognized that these characteristics may also depend on other factors such as age or ethnicity [17–19]. Therefore, further research is needed in different settings to identify the factors that may contribute to gender differences in self-reporting and weight loss outcomes.

### Limitations

This study has limitations that may inspire future research. First, analysis results cannot be free from errors, as the data are self-reported. Particularly, the weight statistics used to divide participants into 3 weight loss groups were self-reported. Although reporting of actual information was encouraged in the research agreements, potential mistakes could have arisen for which participants could have been unaware while reporting their weight. Furthermore, scales that the participants used may not have been reliable. To prevent these potential problems, some weight values were modified by applying logic during preprocessing. However, the applied logic was empirical and somewhat arbitrary. In addition, all reported data other than weight information, such as gender and age, were assumed to be true and consistent throughout the study. No correction was made for misreported data. Further research should be conducted in more sophisticated environments by collecting data under rigorous verification by researchers or health professionals.

Second, there is a potential limitation related to the approach and data analyses in this study. First, the significance of the *P* value may have been underpowered. In particular, only 8.7% (19/218) of the male participants lost more than 10% of their weight (Table 1). Further, only 9.5% (17/178) of the participants who were obese before they started the experiment lost more than 10% of their weight (Table 1). The small sample size could have potentially undermined statistical significance [54]. Second, the interval division criteria applied to the analysis of BMI and BMI delta values ​​are somehow arbitrary. Although methodologies exist for determining trend-change points, such methodologies may not provide adequate solutions in short-term multivariate time-series environments (ie, 16 weeks with 6 series) [55]. Thus, a more rigorous data analysis setting should be established by collecting more data and applying sophisticated approaches.

Third, there may be potential limitations in the experimental setting assumptions. For instance, although the registered dietitians in the program did not recommend the use of weight-reducing drugs like diuretics, some participants might have relied on some medicines that have a huge effect on weight loss. Furthermore, the study design did not include a control group, which might have undermined the ability to evaluate the efficacy of the mobile weight loss intervention. For example, the weights of the subjects who canceled the program prior to week 13 were not analyzed. By analyzing such data in a control group, the efficacy of the mobile intervention can be evaluated in a more rigorous manner. In addition, significant differences in food intake reporting by weight loss outcomes in female participants may have been caused by the Hawthorne effect [56]. In other words, the significant difference may be the result of a keen sense of being observed in the experiment rather than gender. Therefore, further experimentations with more sophisticated settings are essential.

Fourth, two factors may have undermined the representativeness of self-reported data for measurements. First, the number of self-reported entries may not have measured spontaneous adherence levels, because the app provides push notifications to remind users to report their data. Additionally, as the degree of self-reporting decreases over time, the data may be insufficient for representing weight status. Particularly, the average number of weight entries in the 16th week for the lowest weight loss group (<5%) was less than 1.5 per participant. When body weight is reported only once per week, potentially biased values may have been reported depending on the timing of the report (eg, after exercise or immediately after main meals, mornings versus evenings). Therefore, for studies requiring self-reporting, further discussion is required regarding data collection and analytical approaches.

### Conclusions

Mobile technology has increased the convenience of self-reporting when dieting [6,13]. However, it should be noted that technology is not the essence of weight loss; rather, it provides a stimulus through simplified self-reporting that may have positive effects on weight loss. Therefore, further research should be conducted to determine ways to couple mobile technology with human nature to foster more effective dieting and consistently healthy lifestyles.

## Appendix

Multimedia Appendix 1 Food-type criteria according to caloric density.

Multimedia Appendix 2 P values from the ANOVA tests comparing the differences between groups for 16 weeks.

Multimedia Appendix 3 P values from the ANOVA tests comparing the differences in intake amount by food group (green, yellow, and red) in women for 16 weeks.

## Abbreviations

ANOVA: analysis of variance.
