# Supplementary material for: Understanding Time Series Patterns of Weight and Meal History Reports in Mobile Weight Loss Intervention Programs: Data-Driven Analysis
Source: J Med Internet Res. 2020 Aug 11;22(8):e17521. doi: 10.2196/17521 (PMC7448179; doi:10.2196/17521)
Supplement: Multimedia Appendix 1 [file jmir_v22i8e17521_app1.pdf]

Table A1. Food-type criteria according to caloric density

Food Type	Green	Yellow	Red
Solid Foods	CD ≤ 1.0	1.0 < CD ≤ 2.4	2.4 < CD
Beverages	CD ≤ 0.4	0.4 < CD ≤ 0.5	0.5 < CD
Soups/Sauces/Spreads	CD ≤ 0.5	0.5 < CD ≤ 1.0	1.0 < CD
Water, Plain Tea/Coffee, Spices, Vitamin, and Health Supplements	No color	No color	No color

CD stands for caloric density and is calculated by dividing calories by grams or mL (per serving).
