# Supplementary material for: Understanding Time Series Patterns of Weight and Meal History Reports in Mobile Weight Loss Intervention Programs: Data-Driven Analysis
Source: J Med Internet Res. 2020 Aug 11;22(8):e17521. doi: 10.2196/17521 (PMC7448179; doi:10.2196/17521)
Supplement: Multimedia Appendix 2 [file jmir_v22i8e17521_app2.pdf]

Table A2. p-values from ANOVA tests of value difference by groups for 16 weeks

Group \ WeekTest	1	2	3	4	5	6	7	8	9	10	11	12	13	14	15	16
The Number of Weight Entries																
Total[a]	0.05	<0.01	<0.01	<0.01	<0.01	<0.01	<0.01	<0.01	<0.01	<0.01	<0.01	<0.01	<0.01	<0.01	0.05	<0.01
Groups by men[b]	0.72	0.12	0.26	0.12	0.04	0.03	0.34	0.19	0.12	0.098	0.62	0.62	0.85	0.23	0.72	0.47
Groups by women[c]	0.01	0.01	<0.01	<0.01	<0.01	<0.01	<0.01	<0.01	<0.01	<0.01	<0.01	<0.01	0.01	0.11	0.01	0.01
BMI Values																
Total[a]	0.8	0.36	0.54	0.28	0.06	0.07	0.02	<0.01	<0.01	<0.01	<0.01	<0.01	<0.01	<0.01	<0.01	<0.01
Groups by men[b]	0.95	0.75	0.62	0.42	0.49	0.21	0.12	0.09	0.06	0.35	0.12	0.02	0.02	0.02	0.04	0.06
Groups by women[c]	0.75	0.48	0.76	0.63	0.098	0.41	0.24	<0.01	0.04	0.05	<0.01	<0.01	<0.01	<0.01	<0.01	<0.01
BMI Delta[A]																
Total[a]	<0.01	<0.01	<0.01	0.09	0.01	<0.01	0.08	0.01	0.08	0.17	0.01	0.08	0.42	0.28	0.92	0.78
Groups by men[b]	0.32	0.05	0.13	0.3	0.61	0.25	0.81	0.37	0.32	0.18	0.86	0.5	0.37	0.86	0.38	0.61
Groups by women[c]	0.01	0.01	0.03	0.34	<0.01	0.01	0.099	0.06	0.27	0.36	<0.01	0.05	0.61	0.34	0.97	0.98
The Number of Meal History Entries																
Total[a]	0.15	0.06	0.08	0.02	0.05	<0.01	0.01	0.01	0.01	0.01	0.02	0.01	0.08	0.03	0.051	0.02
Groups by men[b]	0.81	0.34	0.37	0.43	0.39	0.32	0.86	0.6	0.75	0.89	0.86	0.86	0.82	0.79	0.72	0.75
Groups by women[c]	0.2	0.28	0.3	0.07	0.09	0.01	0.01	0.02	0.02	0.01	0.01	<0.01	0.099	0.05	0.05	0.03

BMI Delta[A] is defined as the difference between the maximum and minimum BMI in a week; [a]Tested null hypotheses: ANOVA: Three groups (1. reducing more than 10% 2. reducing between 5% and 10%, and 3. reducing less than 5%) have the same value; [b]Tested null hypotheses: ANOVA: Three groups (1. reducing more than 10%, 2. reducing between 5% and 10%, and 3. reducing less than 5%) in men have the same value; [c]Tested null hypotheses: ANOVA: Three groups (1. reducing more than 10%, 2. reducing between 5% and 10%, and 3 reducing less than 5%) in women have the same value. All p values were adjusted by the false discovery rate. Blue shading indicates cases in which the adjusted p value is lower than 0.05 and green shading indicates cases in which the adjusted p value is between 0.05 and 0.1.
