# Supplementary material for: Understanding Time Series Patterns of Weight and Meal History Reports in Mobile Weight Loss Intervention Programs: Data-Driven Analysis
Source: J Med Internet Res. 2020 Aug 11;22(8):e17521. doi: 10.2196/17521 (PMC7448179; doi:10.2196/17521)
Supplement: Multimedia Appendix 3 [file jmir_v22i8e17521_app3.pdf]

Table A3. p-values from ANOVA test results of intake-amount-differences by food types (green, yellow, and red) for 16 weeks

| Gender | Food Color | Food Type | W1 | W2 | W3 | W4 | W5 | W6 | W7 | W8 | W9 | W10 | W11 | W12 | W13 | W14 | W15 | W16 |
|---|---|---|---|---|---|---|---|---|---|---|---|---|---|---|---|---|---|---|
| Women | Green | Breakfast | 0.675 | 0.543 | 0.068 | 0.807 | 0.859 | 0.780 | 0.822 | 0.960 | 0.822 | 0.825 | 0.830 | 0.656 | 0.456 | 0.932 | 0.975 | 0.932 |
| Women | Green | Morning_Snack | 0.427 | 0.716 | 0.822 | 0.825 | 0.932 | 0.822 | 0.911 | 0.968 | 0.989 | 0.867 | 0.867 | 0.453 | 0.388 | 0.830 | 0.932 | 0.716 |
| Women | Green | Lunch | 0.522 | 0.949 | 0.867 | 0.944 | 0.869 | 0.515 | 0.549 | 0.683 | 0.777 | 0.332 | 0.932 | 0.719 | 0.740 | 0.777 | 0.209 | 0.637 |
| Women | Green | Afternoon_Snack | 0.777 | 0.626 | 0.949 | 0.968 | 0.956 | 0.949 | 0.610 | 0.730 | 0.409 | 0.956 | 0.603 | 0.295 | 0.719 | 0.989 | 0.625 | 0.731 |
| Women | Green | Dinner | 0.696 | 0.759 | 0.970 | 0.728 | 0.867 | 0.759 | 0.576 | 0.822 | 0.603 | 0.596 | 0.822 | 0.409 | 0.926 | 0.859 | 0.949 | 0.584 |
| Women | Green | Evening_Snack | 0.949 | 0.487 | 0.324 | 0.489 | 0.818 | 0.989 | 0.777 | 0.642 | 0.868 | 0.549 | 0.409 | 0.807 | 0.549 | 0.932 | 0.975 | 0.822 |
| Women | Yellow | Breakfast | 0.960 | 0.610 | 0.822 | 0.932 | 0.192 | 0.540 | 0.949 | 0.324 | 0.429 | 0.113 | 0.082 | 0.314 | 0.756 | 0.314 | 0.429 | 0.375 |
| Women | Yellow | Morning_Snack | 0.295 | 0.640 | 0.944 | 0.456 | 0.767 | 0.807 | 0.411 | 0.989 | 0.932 | 0.932 | 0.868 | 0.674 | 0.277 | 0.913 | 0.926 | 0.859 |
| Women | Yellow | Lunch | 0.209 | 0.130 | 0.208 | 0.014 | 0.731 | 0.149 | 0.453 | 0.355 | 0.014 | 0.209 | 0.014 | 0.014 | 0.355 | 0.048 | 0.913 | 0.626 |
| Women | Yellow | Afternoon_Snack | 0.822 | 0.932 | 0.641 | 0.808 | 0.523 | 0.629 | 0.557 | 0.926 | 0.932 | 0.314 | 0.960 | 0.198 | 0.626 | 0.637 | 0.714 | 0.949 |
| Women | Yellow | Dinner | 0.014 | 0.124 | 0.209 | 0.068 | 0.822 | 0.014 | 0.014 | 0.112 | 0.100 | 0.155 | 0.100 | 0.014 | 0.209 | 0.309 | 0.036 | 0.164 |
| Women | Yellow | Evening_Snack | 0.603 | 0.949 | 0.949 | 0.626 | 0.603 | 0.626 | 0.997 | 0.515 | 0.656 | 0.582 | 0.675 | 0.764 | 0.956 | 0.603 | 0.932 | 0.645 |
| Women | Red | Breakfast | 0.926 | 0.137 | 0.220 | 0.043 | 0.068 | 0.415 | 0.130 | 0.355 | 0.549 | 0.100 | 0.043 | 0.549 | 0.219 | 0.409 | 0.869 | 0.549 |
| Women | Red | Morning_Snack | 0.158 | 0.468 | 0.696 | 0.420 | 0.949 | 0.025 | 0.535 | 0.932 | 0.051 | 0.949 | 0.535 | 0.949 | 0.777 | 0.610 | 0.949 | 0.989 |
| Women | Red | Lunch | 0.465 | 0.112 | 0.014 | 0.043 | 0.074 | 0.014 | 0.058 | 0.014 | 0.100 | 0.082 | 0.051 | 0.014 | 0.112 | 0.113 | 0.355 | 0.048 |
| Women | Red | Afternoon_Snack | 0.669 | 0.014 | 0.100 | 0.219 | 0.675 | 0.807 | 0.383 | 0.295 | 0.637 | 0.603 | 0.549 | 0.014 | 0.306 | 0.489 | 0.494 | 0.641 |
| Women | Red | Dinner | 0.112 | 0.025 | 0.014 | 0.014 | 0.014 | 0.456 | 0.332 | 0.051 | 0.082 | 0.014 | 0.014 | 0.094 | 0.014 | 0.124 | 0.048 | 0.740 |
| Women | Red | Evening_Snack | 0.051 | 0.603 | 0.243 | 0.603 | 0.112 | 0.968 | 0.100 | 0.924 | 0.489 | 0.014 | 0.932 | 0.535 | 0.309 | 0.626 | 0.626 | 0.549 |
| Men | Green | Breakfast | 0.999 | 0.999 | 0.993 | 0.999 | 0.993 | 0.999 | 0.993 | 0.993 | 0.993 | 0.993 | 0.999 | 0.993 | 0.993 | 0.999 | 0.993 | 0.999 |
| Men | Green | Morning_Snack | 0.993 | 0.993 | 0.999 | 0.993 | 0.999 | 0.993 | 0.993 | 0.999 | 0.993 | 0.993 | 0.993 | 0.993 | 0.993 | 0.993 | 0.999 | 0.999 |
| Men | Green | Lunch | 0.999 | 0.993 | 0.993 | 0.993 | 0.993 | 0.993 | 0.999 | 0.999 | 0.993 | 0.993 | 0.999 | 0.993 | 0.999 | 0.999 | 0.993 | 0.993 |
| Men | Green | Afternoon_Snack | 0.993 | 0.993 | 0.993 | 0.993 | 0.993 | 0.999 | 0.576 | 0.999 | 0.993 | 0.993 | 0.993 | 0.993 | 0.993 | 0.999 | 0.999 | 0.993 |
| Men | Green | Dinner | 0.993 | 0.993 | 0.993 | 0.999 | 0.993 | 0.999 | 0.993 | 0.993 | 0.993 | 0.999 | 0.999 | 0.993 | 0.993 | 0.993 | 0.999 | 0.993 |

| | | | | | | | | | | | | | | | | | | |
|---|---|---|---|---|---|---|---|---|---|---|---|---|---|---|---|---|---|---|
| Men | Green | Evening_Snack | 0.999 | 0.993 | 0.993 | 0.999 | 0.993 | 0.993 | 0.993 | 0.993 | 0.993 | 0.993 | 0.993 | 0.993 | 0.993 | 0.999 | 0.993 | 0.999 |
| Men | Yellow | Breakfast | 0.993 | 0.993 | 0.993 | 0.993 | 0.993 | 0.993 | 0.993 | 0.993 | 0.999 | 0.993 | 0.999 | 0.993 | 0.993 | 0.993 | 0.993 | 0.993 |
| Men | Yellow | Morning_Snack | 0.999 | 0.993 | 0.999 | 0.999 | 0.993 | 0.993 | 0.999 | 0.999 | 0.999 | 0.999 | 0.993 | 0.993 | 0.993 | 0.993 | 0.993 | 0.993 |
| Men | Yellow | Lunch | 0.993 | 0.993 | 0.993 | 0.993 | 0.993 | 0.993 | 0.993 | 0.993 | 0.993 | 0.993 | 0.993 | 0.993 | 0.993 | 0.999 | 0.993 | 0.993 |
| Men | Yellow | Afternoon_Snack | 0.993 | 0.993 | 0.993 | 0.993 | 0.993 | 0.993 | 0.993 | 0.999 | 0.999 | 0.999 | 0.993 | 0.993 | 0.993 | 0.993 | 0.999 | 0.999 |
| Men | Yellow | Dinner | 0.993 | 0.993 | 0.993 | 0.993 | 0.993 | 0.993 | 0.993 | 0.993 | 0.993 | 0.999 | 0.993 | 0.999 | 0.993 | 0.993 | 0.999 | 0.993 |
| Men | Yellow | Evening_Snack | 0.993 | 0.993 | 0.999 | 0.993 | 0.993 | 0.993 | 0.999 | 0.993 | 0.993 | 0.999 | 0.993 | 0.993 | 0.999 | 0.993 | 0.993 | 0.999 |
| Men | Red | Breakfast | 0.288 | 0.999 | 0.993 | 0.993 | 0.999 | 0.993 | 0.993 | 0.993 | 0.993 | 0.993 | 0.993 | 0.993 | 0.993 | 0.999 | 0.993 | 0.993 |
| Men | Red | Morning_Snack | 0.993 | 0.999 | 0.993 | 0.999 | 0.993 | 0.993 | 0.999 | 0.999 | 0.993 | 0.993 | 0.993 | 0.993 | 0.993 | 0.993 | 0.993 | 0.993 |
| Men | Red | Lunch | 0.993 | 0.993 | 0.993 | 0.993 | 0.993 | 0.993 | 0.993 | 0.993 | 0.993 | 0.993 | 0.993 | 0.999 | 0.993 | 0.993 | 0.999 | 0.993 |
| Men | Red | Afternoon_Snack | 0.993 | 0.993 | 0.993 | 0.993 | 0.993 | 0.993 | 0.993 | 0.993 | 0.993 | 0.993 | 0.993 | 0.993 | 0.993 | 0.993 | 0.993 | 0.999 |
| Men | Red | Dinner | 0.993 | 0.993 | 0.993 | 0.993 | 0.993 | 0.993 | 0.993 | 0.993 | 0.999 | 0.993 | 0.999 | 0.999 | 0.993 | 0.993 | 0.993 | 0.993 |
| Men | Red | Evening_Snack | 0.999 | 0.993 | 0.993 | 0.999 | 0.993 | 0.993 | 0.993 | 0.993 | 0.993 | 0.999 | 0.993 | 0.999 | 0.993 | 0.999 | 0.993 | 0.993 |

BRE, MOR.S, LUN, AFT.S, DIN, EVE.S represent breakfast, morning snack, lunch, afternoon snack, dinner, and evening snack, respectively; Avga indicates the grouped average of food intake over 16 weeks; Tested null hypotheses, ANOVA: Three groups (1. reducing more than 10% 2. reducing between 5% and 10%, and 3. reducing less than 5%) have the same value; All p values were adjusted by the false discovery rate. Blue shading indicates cases in which the adjusted p value is lower than 0.05 and green shading indicates cases in which the adjusted p value is between 0.05 and 0.1.
